# A review of prospects and current scenarios of biomass co-pyrolysis for water treatment

**DOI:** 10.1007/s13399-022-03011-0

**Published:** 2022-07-12

**Authors:** Shifa Zuhara, Hamish R. Mackey, Tareq Al-Ansari, Gordon McKay

**Affiliations:** 1 Division of Sustainable Development, College of Science and Engineering, Hamad Bin Khalifa University, Qatar Foundation, Doha, Qatar; 2 Division of Engineering Management and Decision Sciences, College of Science and Engineering, Hamad Bin Khalifa University, Qatar Foundation, Doha, Qatar

**Keywords:** Co-pyrolysis, Biomass, Activation, Water treatment, Adsorption

## Abstract

With ever-growing population comes an increase in waste and wastewater generated. There is ongoing research to not only reduce the waste but also to increase its value commercially. One method is pyrolysis, a process that converts wastes, at temperatures usually above 300 °C in a pyrolysis unit, to carbon-rich biochars among with other useful products. These chars are known to be beneficial as they can be used for water treatment applications; certain studies also reveal improvements in the biochar quality especially on the surface area and pore volume by imparting thermal and chemical activation methods, which eventually improves the uptake of pollutants during the removal of inorganic and organic contaminants in water. Research based on single waste valorisation into biochar applications for water treatment has been extended and applied to the pyrolysis of two or more feedstocks, termed co-pyrolysis, and its implementation for water treatment. The co-pyrolysis research mainly covers activation, applications, predictive calculations, and modelling studies, including isotherm, kinetic, and thermodynamic adsorption analyses. This paper focuses on the copyrolysis biochar production studies for activated adsorbents, adsorption mechanisms, pollutant removal capacities, regeneration, and real water treatment studies to understand the implementation of these co-pyrolyzed chars in water treatment applications. Finally, some prospects to identify the future progress and opportunities in this area of research are also described. This review provides a way to manage solid waste in a sustainable manner, while developing materials that can be utilized for water treatment, providing a double target approach to pollution management.

## Introduction

Environmentally sustainable water treatment options are increasingly being researched and implemented these days to combat the projected water scarcity. The global wastewater production is currently 380 billion cubic meters annually across the world, which is about five-fold times the volume of water moving through Niagara fall every year. This volume is projected to rise by 24% in the coming nine years and 51% by 2050 [1]. There are a number of technologies used to treat this water globally—this includes biological (aerobic and anaerobic treatments using microorganisms), chemical (ozonation, electrochemical, photo-electrochemical, Fenton's oxidation), and physical (adsorption, membrane filtration, coagulation/flocculation) methods [2]. Since there are health and environmental concerns about using treated wastewater due to the detrimental effects of the heavy metal, cations, anions (such as phosphate and nitrate), organics (emerging pollutants – pharmaceuticals, pesticides, endocrine disrupting compounds) and pathogens present in the wastewater; there is an increasing focus on removing these harmful substances. This paper will evolve around discussing the treatment method of adsorption, which is known to be a readily accessible technology, simple to execute, inexpensive, non-destructive, with a remarkable ability to remove pollutants [3].

Consequently, the increasing world population poses a threat to the public due to growing municipal solid waste (MSW) generated, consumption of resources, and the continuing increasing demand for water. Around the world, organic waste, including both food and green waste, forms the most significant component in MSW (around 44%). This is followed by paper and cardboard (17%), plastic (12%), glass (5%), metal (4%), wood (2%), rubber and leather (2%), and other wastes generated at about 14%. It is estimated that there is an increase of 15 million tonnes (Mt) of MSW added to the waste market annually [4]. The total worldwide amount of MSW generated is expected to reach 3400 Mt by 2050; about 12% of MSW produced globally in 2016 was composed of plastics [3]. Hence, sustainable management of MSW is critical for future environmental sustainability.

The most traditional and common treatments for MSW involve open dumping, landfilling, and incineration. All of these methods are associated with numerous environmental risks [4]: incineration causes air pollution and ash disposal after burning; landfilling often leads to leaching causing pollution in surface and groundwater sources and emissions in addition the release of landfill gases (methane and carbon dioxide) to the atmosphere, which is also common in open dumping [4]. Experts revealed that up to 20% greenhouse gas emissions are related to solid waste management activities from developing countries alone. Due to the above-mentioned reasons, alternative methods of effective waste usage need to be considered as part of implementing better and more sustainable practices [4].

In addition to controlling the amount waste produced, recycling opportunities for municipal solid waste management (including food, plastic, and other wastes) should also be explored [5]. Although these techniques are being used to manage wastes, there is still a lack of policy recommendations on the circular economy solution approach, namely, closing loops for all waste streams that a country produces [1]. Sweden is an example where all types of waste are converted to value-added products like biogas, electricity, and heat, thereby reducing the landfilled wastes significantly [6]. Usually, thermochemical conversion processing is considered to convert wastes into useful products. These conversion processes include combustion, gasification, pyrolysis, and liquefaction methods [7]. Pyrolysis converts wastes at temperatures usually above 300 °C in a pyrolysis unit to bio-oil, carbon-rich biochar, and combustible gases [8]. It is being studied on different levels, including micro-level experimental studies to understand the degradation kinetics and to develop more realistic pilot and semi-industrial scale studies to further understand the properties of the biochar-derived adsorbents produced [9]. The pyrolysis conditions play a significant role in the degradation behavior, process efficiency, and cost-effectiveness [9]. Chars and the gases are often considered byproducts from pyrolysis, while the bio-oil portion is the main product. Generally, a low temperature with a slow heating rate leads to maximum char production, and high temperature with long residence time is known to maximize gas production [10].

The feasibility of using biochar in water treatment has been reported in literature [11–13], providing a suitable reuse application. One such study, based on using groundnut, coconut shell, and rice husk for dye removal revealed groundnut to be the most economical route as it was more readily available and cheaper [14]; therefore, low-cost adsorbents are being increasingly researched, especially for large-scale level applications. An interesting study on the industrial application of low-cost adsorbents for dye removal revealed that good accessibility and durability of adsorbents among other factors play a key role in the feasibility of the application. Furthermore, the study even concluded that with good regeneration capabilities and large reuse times, the technology could cost much less than distillation for dye wastewater treatment [15].

However, there is limited implementation on drinking water and wastewater applications and associated policies. The reasons for this are speculated to be inadequate systematic studies and the lack of optimization studies of design/operating parameters [16]. Furthermore, due to the lack of support in technological advancements in some countries in Asia and Africa, there needs to be an improvement in related policy frameworks to enhance research and development in the field [16, 17]. Given the ability of pyrolysis to process different wastes together [18], there is a need for related policy reforms that would enable use of these waste-derived materials in sensitive applications such as drinking water treatment, following appropriate safety demonstration under strict testing protocols.

The vast majority of research and development in biochar-based adsorbents consider a single waste fraction that does not interconnect with MSW well. Of the studies that have considered copyrolysis, which is the pyrolysis of two or more feedstocks, they conclude that co-pyrolysed biochar is an economically feasible option [19] compared to other waste treatment options [20]; and that the added benefits from resource recovery and recycling are known to increase the economic feasibility and process attractiveness [21]. Therefore, in this paper, the potential applicability of co-pyrolysis to produce water adsorbents will be reviewed and assessed. This process represents the real-life scenario of MSW and its applicability—therefore, decreasing the quantity of various waste types at once and reducing pollution significantly.

This paper provides an update on earlier reviews [22, 23] by firstly giving coverage to the most recent works from 2019 onwards and secondly implementing a methodological process focusing on steps and techniques of the production of co-pyrolyzed chars and subsequent water treatment rather than focusing primarily on the feedstocks and/or pollutants. This includes a more comprehensive by report of both experimental and modelling research related to water treatment using co-pyrolysis char, inclusion of activation methods for upgrading co-pyrolyzed chars, information on thermodynamic analysis, regeneration data, and reports for real water studies. This review, therefore, provides a timely and comprehensive reflection on recent work in adsorbents produced from co-pyrolyzed wastes and their application.

**Fig. 1:**
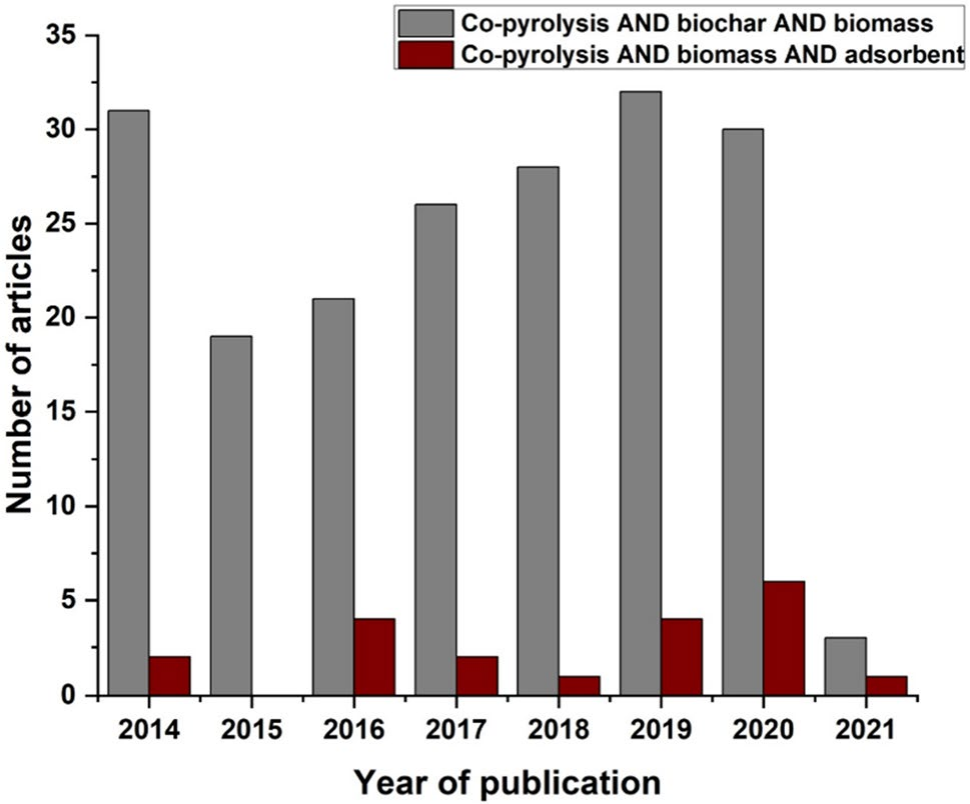
SCOPUS results for co-pyrolysis papers in literature

Among the research articles published so far, this chapter will mainly consider papers published in the past five years (from 2017). The first set of search words are ‘co-pyrolysis’ and ‘biochar’ and ‘biomass’ and the second set of words are ‘co-pyrolysis’ and ‘biomass’ and ‘adsorbent’—these were entered into the Google Scholar and SCOPUS search engines; while the former input search words provided 4300 search results, the latter only gave 2020 results. Figure 1 shows the SCOPUS search in both areas published through the years 2014 to 2021. The papers for both searches fell below the 35 articles/year limit over the years showing that the research is still rising, especially for water treatment applications. The following sections only cover the papers that passed additional sieving and scrutiny based on their alignment with the topic (and search words).

Section 2 will discuss the production of chars that have the potential for water treatment. The succeeding sections will include papers already published on using co-pyrolyzed chars for water treatment involving the treatment of both organic and inorganic pollutants.

**Fig. 2:**
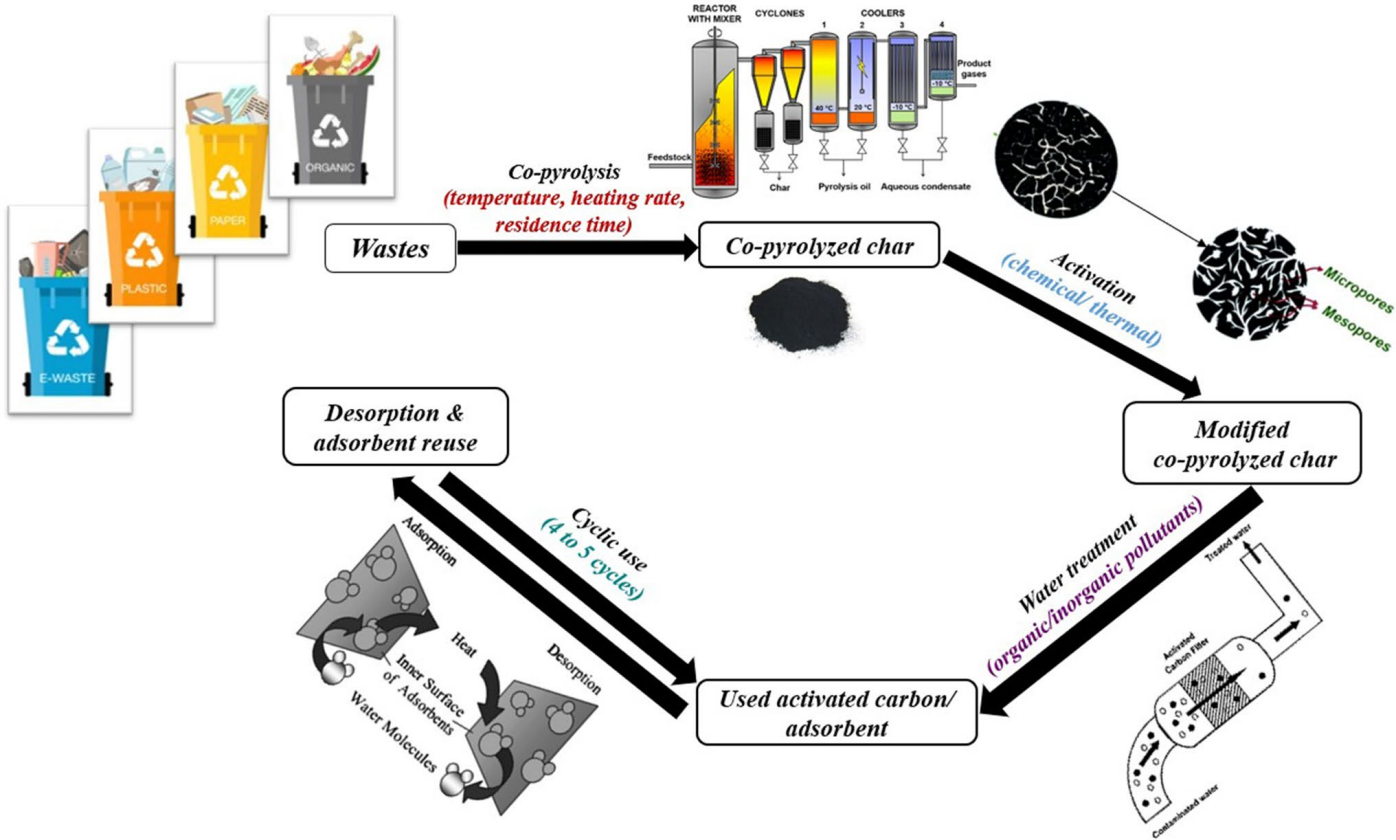
Schematic diagram of wastes to adsorbents for water treatment

The objectives of this paper are (i) to summarize the co-pyrolysis operating conditions and requirements to produce quality char that can be potentially used for water treatment, (ii) to understand various activation techniques for upgrading co-pyrolyzed chars to improve their water pollutant adsorption characteristics, (iii) to discuss water treatment of both organic and inorganic pollutants using co-pyrolyzed char in detail, including experimental parameters (pH, temperature, dose, time), modelling studies (isotherm modelling and kinetic modelling), temperature and thermodynamic analysis, removal mechanisms, regeneration, and real water studies, and (iv) discuss future prospects of water treatment using co-pyrolyzed chars. Figure 2 shows the steps to be followed for converting wastes to co-pyrolyzed chars to activated chars/carbon, which are then used for water treatment and further desorption and regeneration studies to help reuse the adsorbents and recover valuable adsorbed species.

## Co-pyrolysis and potential chars for water treatment

Co-pyrolysis of more than one waste is generally considered useful for a number of reasons apart from reducing the quantity of waste and reducing associated pollution. When compared to single wastes, co-pyrolysis is known to increase the carbon conversion efficiency and the yield of volatiles [24]. Additionally, it is known to have stronger products that individual feedstocks after pyrolysis with better the biochar yield with enhanced properties [22]. The products from this process are also known to be advantageous for water treatment application; a review paper suggests better removal rates for pollutants when using co-pyrolyzed char instead of single waste chars. This can be attributed to the positive synergistic effect observed when co-pyrolyzing wastes, which is highly dependent on the kind of feedstocks used, the pyrolysis operating conditions, and the blending ratio [25].

There are numerous papers published on co-pyrolysis with biomass describing the quality of the char along with operating conditions and feedstock composition. The studies focus on thermogravimetric analysis (TGA) reactors, the tube furnace, fixed bed reactors, agitated bed pyrolysis reactors, the pyrolysis furnace design and in some cases, semi-industrial scale pyrolysis units. One study co-pyrolyzing coal and walnut shells revealed the experimental results for char yield to be higher than the calculated values showing excellent potential for application [26]. Another specific study focused on co-pyrolysis of sewage sludge (SS) with bamboo sawdust, exhausted tea, and rice husk, all giving chars with good yields greater than 50% and a surface area greater than 14 m^2^/g in all cases showing promising potential for activation and further use as an adsorbent [27]. There is research on understanding the effect of mineral components in sludge on the pyrolysis process. One such study, based on TG-FTIR-GC/MS analysis, revealed that the presence of mineral components catalyzed the thermal cracking of the organic components present in the sludge and accelerated the thermal degradation to the value-added products. Specifically, the yield, surface area, and pore structure was known to improve with the inherent minerals present in the sludge samples due to the improvements in metal catalyzed thermochemical reactions during pyrolysis [28]. Furthermore, another study concluded that the increase in pyrolysis temperature is known to increase surface area and stabilize the toxic metals present hence reducing the likelihood of leaching [29]. Hence, the produced char is considered low risk for the environment due to less bioavailability and decreased leaching capacity [30]. This also proves the importance of operating parameters in developing varying quality chars, which includes factors such as temperature, heating rate, operating reactor type, blending ratio, and feedstock types [31]. However, this also highlights that leaching tests or metal-content guidelines are important for the use of the co-pyrolysed biochars in water treatment applications.

There are studies that focus on the interaction, mechanism, and synergy of co-pyrolysis while adding different types of feedstocks. One study on using cellulose and polyethylene was found to lead to CH formation leading to an improvement in fuel products; further studies on molecular dynamic simulation and density functional theory enhanced the production of fuels of high quality [32]. One study used a multi-technology approach to understand the synergistic effects of co-pyrolysis of seaweed and rice husk and concluded that deoxidization effects changed the products distribution [33]. Reaction force field simulation studies have also enabled a better understanding of the interactions in co-pyrolysis to be achieved; a study revealed synergistic effects between rice husk and oily sludge (due to the presence of hydroxyl and hydrogen radicals) with reduced activation energy, even contributing to a difference in product distribution [34]. More specifically, Section 4.3 will discuss the studies investigating the mechanisms of water treatment using co-pyrolyzed chars. Table 1 focuses on 12 co-pyrolysis studies along with varying process conditions, which can be beneficially utilized, upgraded, and applied for water treatment applications.

**Table 1:** Selected co-pyrolysis articles from 2021 focusing on char production

Feedstock	Reactor	Operating condition	Findings/char characteristics	References
Cellulose (C), lignin (L), sawdust (S)	TGA-FTIR	BR: 1:1 T: 500 °C	Lignin enhanced the formation of biochar Yield: S:C-17.5% S: L-41.0% C: L-33.8%	[35]
Enteromorpha prolifera-corn straw	TGA	BR: 7:3, 1:1, 3:7 of corn straw T: 5 °C–600 °C HR: 10 °C/min	BET (m^2^/g): 1.79, 1.41, 0.86 Yield: 35.44, 38.17, 41.76	[36]
Wood sawdust (RWS)-SS (SS)	Agitated bed pyrolysis reactor	BR: (25:75, 50:50, and 75:25 by weight) T: 450, 500, and 550 °C GFR: 5 L/min of N_2_	Improved yield of biochar products with the SS biochar and increasing its carbon content and reducing ash and inorganic elements. RWS biochar surface more porous but SS biochar has larger specific surface Highest yield – RWS 25: SS75—~ 44.5 at 450 °C	[37]
SS and LDPE	TGA	BR: 1:1 and 1:2	SS and LDPE lead to lower formation of char due to synergic interaction between them	[38]
Biomass and bentonite (BBC) with Zn, Fe, and Mn	TGA	BR: 10:1 (CS: bentonite) HR: 10 °C/min	BBC; ZnBBC; FeBBC; MnBBC Yield (%): 39.65; 56.10; 46.93; 49.66 BET(m^2^/g): 70.81; 24.24; 9.24; 37.11	[39]
Low-rank coal (LRC) with lignin (LIG)	Tube furnace	T: 550 – 700 °C BR: 0 -100 wt.%	Highest char yield decreasing from 0 to 100% if coal The char yield of LRC decreased from 80.82 wt.% to 71.51 wt.% from 550 °C to 700 °C, while LIG decreased from 50.55 wt.% to 40.62 wt.%. Mixing both also showed similar behavior	[40]
Pine bark and wheat straw with Tetra Pak waste (TPW)	TGA	HR: 10 C/min T: 25 °C to 700 °C GFR: 40 mL/min of air	Total yield: decreased from 36 wt.% to ~ 18 wt.% for PB-TPW; decreased from 26 wt.% to ~ 18 wt.% from 0 to 100 mass% of TPW Carbon and hydrogen distribution in char yield highest: ~ 60 mass% and ~ 20% at 0:100 (TPW: PB); ~ 40 mass% and ~ 10 mass% at 0:100 (TPW:WS)	[41]
Eucalyptus wood (EW) and LDPE	Semi industrial pyrolysis unit	T: 300 to 550 °C, RT: 90–150 min BR: 33% and 25% of LDPE	Yield %—Highest at 300 °C for 90 min at ~ 35 and ~ 37% for 1:2 and 1:3 feedstock ratio (LDPE: EW) Energy density (1.25) and high heat value (31 MJ/Kg) at 300 °C The highest concentrations of fixed carbon (39%), fuel ratio (0.81) along with the lowest O/C and H/C ratios (0.07 and 0.13) above 450 °C	[42]
Eucalyptus biomass and waste expanded polystyrene (WPS)	Semi industrial pyrolysis unit	T: 300—550 °C RT: 90–50 min BR: (33% and 25%) WPS (w/w)	33% WPS content at 300 °C → Energy density (1.12–1.30), heating value (28.03–32.5 MJ/kg 25% WPS content at 550 C → Fixed carbon (4.5–34.19%), fuel ratio (0.05–0.64) The chars produced at 300, 350 °C were observed to have O/C and H/C ratios like that of sub-bituminous and bituminous coal	[43]
Biomass and single-use plastics	TGA	TGA: T: 300, 400, 500, and 600 °C BR: 2:1 (EW: PS) 2:1 (w/w), EW: LDPE 2:1 (w/w) GFR: 30 mL/min of N_2_ HR: 10 °C/min	Adding PS exhibited highest synergistic and inhibitory effects. After the complete degradation of plastics, char had higher values of surface area (15–64%), and cation exchange capacity (5–19%)	[44]
Corn cob (CC) and PE	TGA	BR: 3:1 HR: 10, 20, and 30 °C/min	AE for CC pyrolysis was estimated to be 240 ± 51.25 kJ/mol Co-pyrolysis required 10% less AE than pyrolysis of CC alone and 50% decrease in bio-char yield for the blend as CC	[45]
SSB (SS blend) with bamboo sawdust (BS), exhausted tea (ET), (KW), rice husk (RH), (WS)	Single step pyrolysis	T: 350–750 °C BR: 4:1 (S/various OF MSW) HR:10 °C min/1 GFR: 100 ml/min of N_2_	Yield; (%) specific surface area (m^2^/g) SSB-BS—59.68 ± 0.85; 20.36 SSB-ET—56.45 ± 1.07; 22.16 SSB-KW—57.37 ± 1.33; 12.11 SSB-PVC—57.20 ± 0.22; 2.20 SSB-RH—58.38 ± 0.77; 16.07 SSB-WS—56.51 ± 0.59; 14.71	[27]
Plastic processing sludge (PPS) and KH_2_PO_4_	Tube furnace with a quartz reactor	BR: KH_2_PO_4_ of 0, 5, 10 and 20 wt.% (KBC-0, 5, 10 and 20) T: 25 – 400 ◦C HR: 10 ◦C/min, final RT: 60 min GFR: 0.5 L/min flow of N_2_	Yield; Surface area (cm^2^ cm ^−3^) PPS: n/a; 1365 KBC-0: 61.13; 1464 KBC-5: 64.26; 1417 KBC-10: 64.77; 1248 KBC-20: 66.02; 1067	[46]

*HR*, heating rate; *BR*, blending ratio; *GFR*, gas flow rate; *T*, temperature; *TGA*, thermogravimetric analysis; *RT*, residence time

## Activation of co-pyrolyzed chars

The production of activated char/carbon involves two main steps: carbonization and activation. The carbonization process removes the volatile content of raw materials when they undergo co-pyrolysis. However, the product, after carbonization, only provides carbons with a high fixed carbon and considerable porosity [47]. Therefore, to improve some of the properties like active sites and functionality, the surface area, and pore volume, activation is carried out. In this process, new pores are created or existing pores are expanded, in addition to changing some other chemical characteristics [48]. These changes make activation a desirable step when the end-use is for water treatment applications.

There are mainly two types of activation methods to prepare activated chars- physical and chemical [49]. Physical methods include oxidizing atmospheric gases such as steam, carbon dioxide, or nitrogen, and in some cases air with temperature ranging from 800 to 1100 °C [49] and chemical activation (wet oxidation) commonly used for biomass activation involves the usage of chemicals in the presence of high temperature. This section will discuss studies that activated co-pyrolyzed chars using these methods.

As mentioned in Section 2, the mineral contents in char samples are known to be stable upon pyrolysis at higher temperature. Specifically, there are recorded effects on activated carbon properties based on the mineral content present in the feedstocks [50]. A study on coconut shell-based activated carbons concluded that the increase in mineral concentration leads to more site occupation of the pore surfaces, which eventually reduces adsorption capacity of the activated carbon [51]. However, another study using brown coals with high mineral content showed good adsorption capacities for pollutants like methylene blue and inorganic pollutants and other gas contaminants [52]. Furthermore, a similar study revealed increased mesoporous properties and reduced surface area in the presence of higher silicon, iron, and calcium using coal-derived activated carbon [53]. One study investigating municipal sewage sludge samples concluded that the risk levels of minerals content are lower after activation with KOH. Sludge samples are known to have varying contents of minerals and therefore such studies are sparse due to the material complexity and variability [54]. Nevertheless, the effect of mineral content on co-pyrolyzed chars has not been extensively reported in literature.

### Physical activation

Generally, physical activation is known to increase the surface and porosity and remove the volatile organic compounds present [55]. Literature on the physical activation of co-pyrolyzed char is much less compared to chemical activation. A study was conducted on activated chars produced from co-pyrolyzed rice husk and polyethylene in equal proportions. Out of the four physical activating conditions in CO_2_, the best textural properties were revealed by the char activated at 800 °C for 4 h exhibiting a surface area of 325 m^2^/g and pore volume of 0.18 cm^3^/g [56]. Another study focused on activating ligneous and herbaceous biomass chars physically with CO_2_ in different reactor types. Results showed an 80% increase in surface area after activation in a mechanically fluidized reactor (MFR) [57]. A similar study on simulated MSW in a CO_2_ environment showed promising results (SA > 300 m^2^/g) at 600 °C and 900 °C [12]. A study on steam activation of char of lignin and ferrous salts showed a relatively low mass-loss rate as the iron oxidized to magnetite in the presence of steam [58].

### Chemical activation

Chemical activation can be accomplished by using alkali, acid, or some neutral activating agents. Chemical activation is known to increase the porosity, functionality, and activated carbon content carried out by the added dehydration agents that enable pyrolytic decomposition, preventing bitumen formation and further thermal degradation [49]. Acid treatment is known to introduce carboxyl groups to the surface. On the other hand, alkaline treatment is known to increase the hydroxyl groups at the surface and to decrease polarization due to the formation of a positive surface charge [55]. Oxidant oxidation is also considered a type of chemical treatment wherein oxygen containing groups is added introducing carboxyl groups onto the surface upon activation in addition to increasing the surface area and porosity [55]. The following sections will discuss studies related to these activation techniques.

#### Acid activation

Several activating chemicals have been tested, but acid activating chemicals mainly include phosphoric and sulfuric acid [49]. A study using sulfuric acid as an activating agent activated co-pyrolyzed chars of sucrose and red mud producing magnetic activated char attaining a maximum surface area of 428 m^2^/g and a pore volume of 0.412 cm^3^/g at 700 °C [59]. Alternatively, using hydrochloric acid for activating peanut shell and vinasse mixtures, the surface area surpassed 1200 m^2^/g and with a pore diameter of 21 Å [60]. A rather unique study focused on using acidic iron salt activating agent (ferric nitrate) after co-pyrolysis of wood and polyvinyl chloride (PVC), demonstrating a moderate surface area of 81.51 m^2^/g and a pore volume of 18.73 cm^3^/g [61].

#### Alkaline activation

Some alkali chemicals used for activation are KOH, NaOH, CaCl_2_, and K_2_CO_3_ [48]. This set of activating agents is possibly the most widely used for activating co-pyrolyzed chars. The first study to be discussed involves a two-step co-pyrolysis treatment of rubber waste and larch sawdust with KOH activation as the second step. The results showed a surface area of 300 m^2^/g was achieved, which was considered of high quality as per the American Water Works Association B600 standard. Additionally, it was also concluded that, the co-pyrolyzed char had a higher surface area compared to the individual ones due to the formation of higher pores after pyrolysis which enables more efficient activation results [62].

A two-fold surface area increase was observed when a sample of sludge and coconut shell co-pyrolyzed char was activated with KOH [63]. There was also a considerable increase in the yield compared to the non-activated chars. Further studies on optimization using the same feedstocks according to a response surface methodology indicated the best yield (47.5%) with 680.3 m^2^/g surface area produced under the conditions listed in Table 2 [64]. Another interesting study with lignin, rice husk, and PVC showed a surface area increase of more than 10 times upon activation with KOH [65].

**Table 2:** Optimized activation condition for co-pyrolysis of coconut shell and sludge [64]

Blending ratio	1:1
Carbonization temperature	500 °C
Carbonization time	45 min
Activating agent (KOH) concentration	2.5 mol/L
Impregnation ratio (KOH solution: sample)	1.5:1
Activation time	60 min
Activation temperature	800 °C

In another experiment, co-pyrolyzed cotton stalk and sludge char were activated with KOH under 5 different reaction conditions [66]; the optimal result for a loading rate of 150 g (30% sludge) was demonstrated by the use of 50% KOH solution, at a radiation time of 24 min at 280 W. Another study performed KOH activation on co-pyrolyzed corn stalk and polyethylene chars, producing a maximum surface area of 581.8 m^2^/g upon activation [67]. Applying KOH in the co-pyrolysis of cyanobacteria and plastics produced a high quality carbon demonstrating a high surface area of 1461 m^2^/g [68].

Potassium carbonate is another common activating agent. One study conducted on co-pyrolyzed municipal sludge and hazelnut shell chars (850 °C) with K_2_CO_3_ under nitrogen atmosphere provided activated chars with a surface area of 1990 m^2^/g and a pore volume of 0.589 cm^3^/g [69]. One more fascinating study on co-pyrolyzed cyanobacteria and plastics showed a surface area of 2135 m^2^/g upon activation with K_2_CO_3_ [70]. A study on the activation of SS and cotton stalks concluded that there was more than a double increase in the surface area when activated with 10 wt% potassium carbonate [71].

#### Neutral activation

Some research studies also use neutral activating agents such as ZnCl_2_. This section will cover two such studies. The first study investigated the activation of co-pyrolyzed chars of municipal sewage sludge and hazelnut shells with ZnCl_2_ giving a surface area of 598.7 m^2^/g. This study also revealed that the ecological risk of heavy metals is very low in chars produced at 500 °C [72]. Another study utilized mixed date stones/pits and olive stones for char production; the surface area increased to 936.5 m^2^/g and the pore volume to 0.593 cm^3^/g after activation with ZnCl_2_ [73].

#### Oxidation activation

Some researchers include chemicals like phosphoric acid, nitric acid, and potassium hydroxide as oxidizing agents. These agents are known to produce oxygen containing functional groups such as –OH, –COOH, -C = O, and -C-O onto the surface of carbon-containing adsorbents. However, this section will include other strong oxidizing agents like hydrogen peroxide and potassium permanganate not mentioned previously in this paper [74]. One of the major oxidizing agents is hydrogen peroxide, which is known to produce free radicals by catalytic conversion. A recent review paper summarizes its use and proposed a mechanism for pyrolyzed biomass samples [75]. One study revealed a surface area of 273.9 m^2^/g when rice stalks were activated with hydrogen peroxide with microwave radiation [76]. Another study added persulfate in addition to hydrogen peroxide as the most frequently used oxidizing agent. However, this agent (like hydrogen peroxide) needs to be activated to generate reactive oxidative species (ROS) which mainly aids in the removal of organic substances [77]. In a special case, dewatered sludge samples with high content of iron-present as Fe_3_O_4_ were revealed to have an advantage with persulfate activation leading to increase in oxygen radicals and porous properties which enabled removal of an azo dye [78]. Potassium permanganate is usually used along with other chemicals such as iron (Fe II) or potassium hydroxide producing chars with good properties [79–81]. Furthermore, there are also some other less studied methods of oxidation like electrochemical, graphite, and plasma treatments [74]. Biochars that are oxidized are known to be excellent for the removal of organic pollutants, the remediation of soil and airborne particulate matters among other applications [82]. Unfortunately, it is uncommon in literature to find co-pyrolysis studies that conduct oxidation activation; therefore, researchers can explore this technique to improve the quality of chars in the coming years.

### Comparison studies

Some studies compare two types of activation methods to examine the difference in the quality of activated chars produced. These provide greater insight into the real potential of an activation method. The subsequent studies use KOH as one of these activating agents. The first study conducted activation by KOH and ZnCl_2_ on chars from co-pyrolyzed waste truck tires and spent tea leaves at 800 °C. The best results were found by zinc chloride activation at a blend ratio of 1:3 (waste truck tires: spent tea leaves), giving a surface area of 527.2 m^2^/g and a pore volume of 0.123 cm^3^/g. It was found that zinc chloride activation produced more carbons with low oxygen content compared to KOH activated chars. However, the quality of both was comparable to those of commercial products [83]. Likewise, thermal activation was compared to KOH activation [84] and H_3_PO_4_ activation [85]. The former study showed a surface area increase of 60% when chemically activated at the same activation temperature, while the latter study showed only a surface area increase of 73 m^2^/g when chemically activated. However, the activation was conducted at different temperatures.

The majority of these activated chars possess good properties, which are considered of great benefit for water treatment. Generally, chemical activation can be regarded as more thoroughly researched and implemented in literature. The next section will discuss in detail studies using co-pyrolyzed chars for water treatment.

## Adsorption studies using co-pyrolyzed chars

In recent years, biochars derived from co-pyrolyzed biomass wastes have been studied and applied to remove both organic and inorganic pollutants from water [13]. One such study revealed a methylene blue removal capacity of 490 mg/g using co-pyrolyzed cyanobacteria and polyethylene wastes (20 mg adsorbent; 298 K; 500 mg/L of methylene blue solution) [70]. Another study achieved 99.5 mg lead/g removal using co-pyrolyzed corn stalk and polyethylene (0.1 g adsorbent; 298.15 K; 50 mg/L; pH 4.5) [67].

### Parameters effecting pollutant adsorption in water

There are several parameters affecting the adsorption process in water. The majority of these factors are described in a recent review paper on adsorption of pollutants [86] including the effects caused due to the operating unit, co-existing ions and other substances, adsorbent particle size, height (in fixed bed systems), feeding flow rate and liquid superficial velocity, feed concentration (in a continuous adsorption system), pH of the solution, adsorption temperature, adsorbent dosage, and contact time. The effect of the latter four parameters on adsorption using co-pyrolyzed chars is discussed in sections from 4.1.1. through 4.1.4, as it is widely reported in literature for co-pyrolysis studies.

The other effects related to adsorption using co-pyrolyzed chars, although less reported, are deemed important. In general, when it comes to the influence of the operating unit, fixed bed adsorption or batch adsorption may be preferred based on the solution concentration and other factors [86]. Furthermore, there are some studies discussing the effect of co-existing pollutants in the adsorption of target pollutants [86]. A study using rice husk with Mg/Al-calcined layered double hydroxides showed a slight reduction (9%) in the phosphate removal in the presence of another ion HCO_3_^−^. The reduction is considered less due to the lower partial negative charges on oxygen atoms on HCO_3_^−^ compared to phosphate ions [87]. More in-depth studies need to be carried out to understand the effect of this factor. As for the particle size and height, it is especially important for fixed-bed column adsorption systems as it helps understand the pressure conditions and related handling enabling good adsorption rates. As a conclusion, increasing the height of the bed is known to improve adsorption as the number of active sites and contact time are increased reducing dispersion [86]. In terms of the feed flow rate, it was noted that the flow rate influences the contact time, residence time, and velocity, which all effects the adsorption rate of pollutants. Similarly, increasing the feed concentration has also been observed to be an essential factor enhancing the adsorption capacities in continuous systems, due to the impact on the mass transfer from the larger concentration gradient between the adsorbent and the solution [86].

#### Effect of pH

Many studies focus on the effect of pH on pollutant removal efficiencies. For example, a study [59] using activated co-pyrolyzed chars of sucrose and red mud chars for chromium removal found a maximum removal capacity, q_e_ ≈ 7 mg/g, at a pH of 2.10. Further reductions in removal rates with pH increase were observed [59]. However, another study [88] focused on utilizing co-pyrolyzed chars of nano-zerovalent iron (nZVI) and SS wastes for the removal of chromium revealed that the lower initial pH is useful for protonation of the biochar surface, hence, promoting chromium removal. At higher pH, chromium (VI) is converted to chromium (III), which is known to be challenging to remove by adsorption [88]. This hypothesis is supported by two other studies. The first one studied co-pyrolyzed char for the removal of arsenic, achieving a maximum adsorption capacity of 4.23 mg/g and removal efficiency of 84.57%, which was obtained at an initial pH of 2 [89]. The second study, using halloysite and coconut shell co-pyrolyzed char, showed optimal lead adsorption at pH 5 [90]. Furthermore, a further study revealed good adsorption results for cadmium at a higher pH using a thiourea modified poplar biochar [91]. Acidic and neutral pH levels were preferred for phenol removal by coal tar pitch and vinasse co-pyrolyzed char, owing to the lower dissociation of phenol at the specified conditions and hence, easier removal. Interestingly, another study revealed negative adsorption of phosphate at pH 2, speculating that phosphate was released from the biochar (SS and walnut). Therefore, adsorption studies focused on pH values greater than 4. Iron-bearing mineral with rice straw biochar composites best-removed chromate (34%) and selenate (37%) between pH 4 and 7 [92]. A similar result was observed in the removal of cadmium by co-pyrolyzed SS and tea waste: the efficiency spiked from 44 to 94% when the pH was increased from 3 to 6 [93].

Another study [92] concluded that it is optimal to conduct pollutant removal from water at higher pH values (greater than 6.0) to avoid precipitation due to protonation at lower pH, which is known to reduce the chances of metal ions binding (the adsorbent used was camellia oleifera shells (COS) with ammonium polyphosphate) [92]. A similar observation was made for the adsorption of basic blue 21 dye by co-pyrolyzed chars of Saccharina japonica and goethite optimized at pH 8 [94]. At low pH, there is competition between the hydrogen ions and the metal/cationic dye ions for the negatively charged adsorbent sites.

#### Effect of temperature

Temperature is known to play a significant role in the uptake of the pollutant, the transformation of surface complexation structures, and also the stability of precipitates if formed [95]. The effect of temperature is widely studied in literature. The first investigation focusing on the influence of temperature was studied for the removal of chromium and arsenic using nZVI and SS. The former study demonstrated an increase in the removal capacities from 11.54 to 12.34 mg/g when the temperature increased from 288 to 318 K [88]. On the other hand, the second study reported an increase in adsorption capacity from 4.42 to 4.88 mg/g of arsenic when the temperatures rose from 298 to 318 K [89]. Another study focused on understanding the effect of increasing the temperature from 573.15 to 873.15 K for the removal of cadmium utilizing a thiourea modified poplar biochar; the results concluded a steady increase in the removal capacity reached 36.53% at 873.15 K [91]. Similarly, a mercury removal study revealed that with an increase in temperature, there is a steady increase in mercury removal efficiency, reaching a maximum of 99% at 32 ºC [96]. The adsorbent used was a co-pyrolyzed bamboo and bromine flame retardant char. Furthermore, another study focusing on rice straw and SS co-pyrolyzed char for the removal of chromium obtained an increase in the metal ion removal capacities with an increase in temperature reaching a maximum adsorption capacity close to 50 mg/g [97].

#### Effect of dose

The dosage of adsorbent is known to play a vital role in the removal of pollutants as the adsorption process depends on the available active sites [93]. One study focused on the dose of co-pyrolyzed char (nZVI and SS) for the removal of chromium and arsenic. For the former, the best removal amount, 13.12 mg/g, was demonstrated when the initial adsorbent dosage was 60 mg/L, and on the other hand, the removal efficiency of the former reduced from 88.92 to 58.00% when the initial dosage increased from 3 to 60 mg/L [56, 57]. A similar study using thiourea modified poplar biochar concluded that the removal capacity increased significantly, with a maximum removal rate of 98.15% when the adsorbent dosage was at 4 g/L [91]. A very close result was obtained with 94% removal of cadmium with 4 g/L biochar (corn stalk and polyethylene) dose [93]. Finally, a study found the highest removal rate (9.23 mg/g) of chromium at an initial pollutant concentration of 50 mg/L using 5 g/L adsorbent [85].

#### Effect of contact time

Usually, contact time studies precede and facilitate kinetic adsorption modelling (Section 3.3.2). A study explained the adsorption removal of chromium by co-pyrolyzed rice straw and SS in two stages- the first, being rapid removal from 0 to 6 h, and the second phase increase being more gradual and slow from 6 to 24 h reaching equilibrium at 24 h and a maximum adsorption capacity of 48.5 mg/g [97]. The two stage rate process occurs in many adsorption systems: the first initial rapid stage is due to adsorption by film diffusion across the boundary layer at the adsorbent surface sites and adsorption into the macro- and large mesopores close to the adsorbent external surface, followed by a slower second stage due to surface and/or pore diffusion into the smaller meso- and micropores in the internal structure of the adsorbent particle [64, 65].

Other studies also follow this trend showing an initial rapid increase in the adsorption capacities with increase in time below 4 h. The adsorption rate then slows down and finally plateaus between 6 and 8 h to a constant saturation capacity value. A maximum lead removal of 97.35 ± 2.73% was obtained using sludge and corncobs (SCB) co-pyrolyzed char at 8 h [98]. Another study on the adsorption of phenol by coal tar pitch and vinasse co-pyrolyzed char equilibrated after 200 min with an adsorption capacity of 36 mg/g [99]. However, another study revealed the adsorption of chromium using co-pyrolyzed sucrose and red waste mud occurred much quicker, within 40 min, and then reached equilibrium after 120 min due to the reduction in the number of available active sites [59].

The following two studies focused on the potential of nZVI and SS co-pyrolyzed char for the removal for chromium and arsenic based on reaction time. Similar to the previous study, the initial removal was rapid (below 2 h), and then the uptake rate decreased progressively as time increased due to the lack of binding sites. Equilibrium was reached with 12.23 mg/g adsorption capacity at 24 h. Consequently, the removal tendencies of arsenic also followed the same pattern, with a maximum adsorption capacity of 4.43 mg/g achieved at 24 h [89].

### Mathematical calculations and modelling

Generally, the calculation and modeling components of water treatment data analysis commonly include isotherms, kinetics, and thermodynamics.

### Isotherm modelling

Isotherm studies are widely used to understand the connection between the adsorbed contaminant and the remainder of the pollutant in water under thermodynamic conditions [100]. The graphs produced along with the curves help to determine the classification of the adsorption systems. Some of the standard models include Freundlich, Langmuir, Redlich-Peterson, SIPS, Toth (Table 3). The following section will discuss different studies utilizing some of these models to understand the adsorption capabilities of co-pyrolyzed chars [100].

**Table 3:** Descriptions of isotherm models

Isotherm model	Description
Langmuir [101]	Considers adsorption as a continuous bombardment of molecules onto a surface with their corresponding desorption or evaporation from the surface with no aggregation at the surface
Freundlich [102]	Not limited to monolayer formation and can be applied to formation of multilayers. Adsorption heat does not need to be uniformly distributed on the heterogeneous surface of the isotherm
Redlich**–**Peterson [103]	Can be applied to heterogenous and homogenous systems as it features both Freundlich and Langmuir models
SIPS or Langmuir–Freundlich (LF) [104]	Combines Langmuir and Freundlich isotherm models to predict the heterogeneity of the system- it localizes the adsorption without adsorbate–adsorbate interaction
Toth [105]	Is a modified version of Langmuir model described for heterogenous system considering both low and high concentration of adsorbate (assuming most sites having lower energy)
Temkin [106]	Considers the interaction between the adsorbent and the adsorbate by ignoring the extremely large and low concentration values- it assumes that adsorption heat of all molecules in the layer declines linearly rather than logarithmically
Dubinin**-**Radushkevich (DR) [107]	Associates the mechanism of adsorption to the distribution of Gaussian energy onto the heterogeneous surfaces

A study that used activated co-pyrolyzed chars of sucrose and red mud for chromium removal focused on both Freundlich and Langmuir isotherm models; the former model produced a higher correlation of the adsorption data with R^2^ = 0.998 than the latter with R^2^ = 0.928 [59]. A similar study focused on these models, and the lead adsorption data using co-pyrolyzed rape straw and orthophosphate showed a higher correlation with the Freundlich model (R^2^ = 0.909) [100]. Another interesting study focused on these models using adsorption data of co-pyrolyzed char of dewatered alum sludge and molasses for arsenic removal in different environments, namely, dry air, nitrogen, and carbon dioxide. Again, the Freundlich isotherm model showed excellent correlation (R^2^ > 0.96) in all studied conditions [108]; similar fit results were concluded using co-pyrolyzed chars with different ratios of rape straw and phosphate rock [109]. This proves that most co-pyrolysis studies provide a better fit to the Freundlich model owing to model’s assumption of heterogenous active sites, which would likely be true in an adsorption system with multiple feedstocks.

Furthermore, a study focusing on removal of methylene blue and congo red using sludge and coconut shell focused on Langmuir and Freundlich isotherms models at three different temperatures (293.15 K, 303.15 K, and 313.15 K). The Langmuir model demonstrated better correlation (R^2^ > 0.97 for all conditions) with the adsorption data [63]. On the other hand, another study focused on the removal of cadmium by co-pyrolyzed SS and tea waste at 298.15 K, 308.15 K, and 318.15 K showing best fit using Freundlich isotherm model in the first two temperatures and the Langmuir model at 318.15 K [63]. Furthermore, according to a study concentrating on using co-pyrolyzed chars of COS and ammonium polyphosphate (produced at varying ratios) for lead removal concluded Langmuir model as the best fit for all conditions [110]. The results of these studies show that adsorption mainly occurred in a monolayer. The reason could be that one of the feedstock dominates the adsorption system or that both feedstocks behave in the same way.

There are some studies that focused on the SIPS (Langmuir–Freundlich) model as well. One such study was conducted at temperatures 283.15 K, 298.15 K, and 313.15 K. The R^2^ value was highest and greater than 0.99 for SIPS in all cases [93]. In another study, involving the application of the Langmuir, Freundlich, and Sips isotherm analyses, the best-fit R^2^ was greater than 0.988 in all chosen temperatures (288.15, 298.15, and 308.15 K) based on the SIPS model [111]. These results show that the SIPS model, which is a hybrid of both Langmuir and Freundlich isotherm models, predicts the heterogeneity of the system very well.

From the previous discussion regarding isotherm types, it is evident that most studies focus on Langmuir and Freundlich models for isotherm modelling. Based on the adsorption data, either one of these models are almost always confirmed as best-fit, without any testing of alternative isotherm models. The Langmuir model usually indicates that the sorption has occurred on a monolayer and has an equal affinity to all active sites on the sorbent surface, on the other hand, a better fit to the Freundlich model implies a heterogeneous sorption system with various active sites [112].

Further information on isotherm models and its application are detailed in Section 3.4.1. Table 4 provides details on 15 studies that focus on isotherm models for removal of some inorganic and organic pollutants.

**Table 4:** Isotherm studies of water treatment using co-pyrolyzed chars

Feedstock	Pollutant	Experiment conditions	Best isotherm model	R^2^/ SSE	Parameter values	Reference
Inorganic pollutant removal studies						
SS and walnut shell	Ammonium Phosphate	Initial pollutant concentration: 50 mg/L Adsorbent amount: 0.1 g Time: 36 h	Freundlich SIPS	0.99 0.981	K_F_ = 0.15605 (mg^1−1/n^⋅L^1/n^⋅g^−1^) n = 1.4605 K_F_ = 0.0078 (L^1/n^/mg^1/n^) n = 2.3179	[113]
nZVIand SS	Arsenic	Initial pollutant concentration: 3–60 mg/L Adsorbent amount: 4 g/L Initial pH: 2–12	Freundlich	0.9950	K_F_ = 1.653 (mg g^−1^ mg^1/n^ L^−1/n^) n = 1.919	[89]
Molasses and dewatered alum sludge	Arsenic	Initial pollutant concentration: 10, 50, 80, 100, 300, 500, 800 and 1000 mg/L Adsorbent amount: 1 Time: 8 h	Freundlich	0.97	K_F_ = 1.08 1/n = 0.48	[108]
Poplar bark and thiourea Poplar saw dust (MB) and thiourea	Cadmium	Initial pollutant concentrations: 5,10, 30, 100, 250, and 500 mg/L Adsorbent amount: 1 g Temperature: 25 ºC	Langmuir Langmuir	0.91 0.99	K_L_ = 0.026 (L/mg) Q_m_ = 19.99 (mg/g) K_L_ = 0.652 (L/mg) Q_m_ = 0.385 (mg/g)	[91]
Cantaloupes straw and polypropylene	Cadmium	Adsorbent amount: 0.02 g Initial pollutant concentration volume: 10 – 400 mg/L Temperature- 25 °C	Langmuir	0.993	K_L_ = 0.25(L/mg) Q_m_ = 108.91 (mg/g)	[114]
SS and hazelnut (magnetized with nanosized γ-Fe2O3)	Copper	Adsorbent amount: 1.25 g/L Initial pollutant concentration: 20 mg/L Temperature- 25 °C	Langmuir	0.995	K_L_ = 0.375(L/mg) Q_m_ = 83.33 (mg/g)	[115]
SS and hazelnut shell	Copper	Initial concentrations: 20, 40, 50, 60, 75, 80, and 100 mg/L Temperature- 25 ◦C	Langmuir	0.999963	K_L_ = 0.62 (L/mg) Q_m_ = 43.54 (mg/g)	[116]
nZVI and SS	Chromium	Initial pollutant concentrations: 20–60 mg/L Adsorbent amount: 4 g/L pH: 2.0 to 6.0	Langmuir	0.9931	K_L_ = 3.953 (L/mg) Q_m_ = 13.27 (mg/g)	[88]
Sucrose with waste red mud	Chromium	Initial pollutant concentrations: 1 to 150 mg/L Adsorbent amount: 0.04 g Temperature: 25 ◦C	Freundlich	0.998	K_F_ = (3.148 mg g^−1^ mg^1/n^ L^−1/n^) n = 1.383 L/mg	[59]
Rape straw and orthophosphate	Lead	Initial pollutant concentrations: 0.05–6 mmol/L Adsorbent amount- 20 mg Temperature- 25 °C pH = 5.0 ± 0.1	Langmuir	0.975	K_L_ = 9.986 (L/mmol) Q_m_ = 1559.3 (mmol/kg)	[117]
Sludge and corncobs (SCB)	Lead	Amount of adsorbent: 0.1 g Initial pollutant concentrations: 10, 20, 40, 60, 80, and 100 mg/L Temperature: 25 ºC	Freundlich SCB (300 °C) SCB (500 °C) SCB (700 °C)	R^2^ > 0.96 to 0.99	K_F_ = 2.2610 (L/mg) 1/n = 0.5288 K_F_ = 2.5540 (L/mg) 1/n = 0.5534 K_F_ = 2.5112 (L/mg) 1/n = 0.5818	[98]
Rape straw and phosphate rock	Lead	Adsorbent amount: 0.1 g Initial pollutant concentration:0 − 5 mmol/L Temperature: 25 °C pH: 5.0 ± 0.1	Freundlich RS: PR (5:1) RS: PR (2:1) RS: PR (1:1)	0.946 0.943 0.965	K_F_ = 144.5 (mmol/kg) n = 0.340 K_F_ = 152.6 (mmol/kg) n = 0.258 K_F_ = 93.96 (mmol/kg) n = 0.312	[109]
Halloysite and coconut shell	Lead	Adsorbent amount: 100 mg Initial pollutant volume: 500 mg/L Temperature- 25 °C	Langmuir	0.9827	K_L_ = 0.018 ± 0.019 (L/mg) Q_m_ = 833.33 ± 16.71 (mg/g)	[90]
Organic pollutant removal studies						
Hematite**-**biochar composite (FOC) Pyrite**-**biochar composite (FSC)	Norfloxacin	Adsorbent amount: 0.1 g Initial pollutant concentrations: 2 mg/L to 30 mg/L Temperature: 15 °C, 25 °C, 35 °C pH: 7.0 ± 0.05	Freundlich FOC (288.15) FOC (298.15) FOC (308.15) FSC (288.15) FSC (298.15) FSC (308.15)	0.988 0.9307 0.9879 0.9872 0.9913 0.9874	K_F_ = 1.8162 (mg^(1−n)^ L^n^/g^−1^) 1/n = 0.2300 K_F_ = 2.1188 (mg^(1−n)^ L^n^/g^−1^) 1/n = 0.1846 K_F_ = 2.4035 (mg^(1−n)^ L^n^/g^−1^) 1/n = 0.1796 K_F_ = 2.9836 (mg^(1−n)^ L^n^/g^−1^) 1/n = 0.2410 K_F_ = 2.9992 (mg^(1−n)^ L^n^/g^−1^) 1/n = 0.2591 K_F_ = , 3.3243(mg^(1−n)^ L^n^/g^−1^) 1/n = 0.2163	[111]
SS and bamboo waste	Ciprofloxacin	Adsorbent amount: 0.25 g Initial pollutant concentrations: 2 mg/L to 30 mg/L Temperature: 30 ºC Time: 24 h pH: 6.0	Freundlich	0.98	K_F_ = 2.55 n = 0.51	[118]
Mixed date pits and olive stones	Dibenzothiophene	Adsorbent amount: 0.1 g Initial pollutant concentrations: 25 − 200 mg/L Temperature: 30 °C	Freundlich	0.9812	K_F_ = 1.29 (mg/g) n = 1.2	[73]
Coal tar pitch and vinasse	Phenol	Adsorbent amount: 0.025 g Initial pollutant concentrations: 10, 20, 30, 50, and 70 mg/L Temperatures: 15 °C, 25 °C, 35 °C	Langmuir 288.15 K 298.15 K 308.15 K	0.997 0.9978 0.9952	K_L_ = 0.12 (L/mg) Q_m_ = 42.7 (mg/g) K_L_ = 0.14 (L/mg) Q_m_ = 47.6 (mg/g) K_L_ = 0.16 (L/mg) Q_m_ = 57.5 (mg/g)	[99]
Corn straw and sawdust Two co-pyrolyzed chars (1:1) at 300º C (BC_300A_) and 800 ºC (BC_800A)_	Atrazine	Adsorbent amount: 10 mg Initial pollutant concentration: 15–55 mg/L Temperature- 25 °C	Freundlich	0.997 0.996	K_F_ = 0.0200 ± 0.00565 (mg/ kg)/(mg/L)^1/n^ 1/n = 1.90 ± 0.0746 K_F_ = 0.00300 ± 0.00167 (mg/ kg)/(mg/L)^1/n^ 1/n = 2.55 ± 0.129	[119]
Saccharina japonica and goethite	Basic blue 41	Adsorbent amount: 10 mg Initial pollutant concentration: 2000 mg/L Temperature: 30 °C	Langmuir	0.983	K_L_ = 0.048 (L/mg) Q_m_ = 803.6 (mg/g)	[94]

#### Kinetic modeling

Kinetic modelling is also essential in designing adsorption systems for water treatment and for batch systems; kinetic curves are usually reproduced as plots of adsorption capacity versus time or contaminant concentration [119]. However, the curve can also be generated in the form of dimensionless contaminant concentration ratio, C_t_/C_0_, in the column outlet and the bed volumes treated in time, t, for fixed-bed studies. This paper will focus on batch adsorption studies following some models, namely, the pseudo-first-order, pseudo-second-order, Avrami, and Elovich, among others (Table 5). The following section will include kinetic batch adsorption studies using co-pyrolyzed char for water treatment, including inorganic and organic contaminants. Table 5 gives an overview summary of when different kinetic or mass transfer models are applicable. The type of mechanism for an adsorption process depends on the nature of the adsorbate relative to the type of surface sites available on the adsorbent. Based on the discussion relating to the application of different types of isotherms, then different adsorption mechanisms may be taking place on the same heterogeneous adsorbent. The chemical/ion exchange sorption processes follow one or more of the following kinetic models: pseudo-first, pseudo-second, Avrami, and Elovich models. For diffusion controlled mechanism processes, these may follow one of the following: Weber-Morris, intraparticle diffusion, Boyd, or Bangham models. Similarly, multi-mechanistic diffusion models have been developed such as the chemisorption-diffusion model, external boundary layer-surface diffusion model, external boundary layer-pore diffusion model, and external boundary layer + pore diffusion + surface diffusion model.

**Table 5:** Descriptions of kinetic models

Kinetic model	Description
Pseudo-first order [120]	Adsorption is the difference equilibrium adsorption and the adsorbed capacity at time multiplied by the rate constant of the adsorption. The rate of adsorption is proportional to this driving force linearly
Pseudo-second order [121]	Adsorption is the difference between the equilibrium adsorption capacity and the adsorbed capacity multiplied by the rate constant. However, in this model, the rate of adsorption is proportional to the square of the driving force indicating each adsorbate occupies two adsorption sites
Elovich [122]	This model looks into this from a chemisorption kinetics perspective by describing the reduction in rate of adsorption due to increase in surface coverage with time
Avrami [123]	This model is adapted from Avrami’s kinetic decomposition model which is used to evaluate the reaction rate as the fraction of adsorption at time, and the rate constant. It also considers multiple adsorption sites
Weber and Morris [124]	The equation for the Weber and Morris intraparticle diffusion model is based on some assumptions. Firstly, it assumes that the resistance to mass transfer is only significant at the beginning of the diffusion. Secondly, the concentration governs the radial diffusion process, only constant diffusion occurs in the process
Diffusion-chemisorption [125]	The diffusion-chemisorption model can be used to describe the sorption of adsorbate onto the heterogeneous surface. The model correlates the rate of change of concentration in solid phase to the rate of mass of transfer of pollutant in fluid phase to the biosorption side
Bangham [126]	It is a logarithmic model used to evaluate the ability of pore diffusion in the adsorption process
Boyd [127]	This model studies if adsorption is taking place by film diffusion or intra-particle diffusion as it assumes that the boundary of the adsorbent has a significant impact on the diffusion of the solute

A study [63] considered the removal of cadmium by utilizing co-pyrolyzed char of SS and tea waste. The kinetic adsorption studies only focused on modeling using the pseudo-second-order model. The results revealed that there was a very good correlation to the adsorption data with an R^2^ value of 0.9. Usually, this model describes a chemisorption process concerning valency forces and ion exchange [63]. The removal of cadmium was also concluded to have taken place using such a mechanism describing the external liquid film diffusion, surface adsorption, and intra-particle diffusion processes accurately.

The following section will discuss studies centered on analyzing data using multiple kinetic adsorption models like pseudo-first order and pseudo-second order models and diffusion mechanistic studies such as the Boyd model and intraparticle diffusion models. The first study focused on three models: pseudo-first-order, pseudo-second-order, and intraparticle diffusion models. The results concluded that the pseudo-second-order model is the most suitable model (R^2^ = 0.99), concluding its suitability for removing chromium using rice straw and SS co-pyrolyzed char [97]. On the other hand, the second study used these three models in addition to the Boyd model to understand the kinetics of phosphate removal using co-pyrolyzed rice husk functionalized with Mg/Al-calcined layered double hydroxides. The results showed that based on R^2^ values, the best models were in the order: pseudo-second order > Boyd > intraparticle diffusion > pseudo-first-order [87]. Hence, it was concluded that the data follows a chemisorption mechanism with possibly multiple layer adsorptions on heterogeneous binding sites. Additionally, the diffusion model results suggest that the initial rate-determining step was the boundary layer film diffusion.

However, in literature, the majority of the studies only focus on comparing pseudo-first and pseudo-second-order models. According to a study of lead adsorption by co-pyrolyzed polyethylene and corn stalk, the rate favored the pseudo-second-order model (R^2^ ≥ 0.9395) over the pseudo-first-order model (R^2^ ≤ 0.7725) [67]. A similar study used co-pyrolyzed rice wastes and polyethylene for the kinetic adsorption of chromium, and in accordance with the previous research, the preferred model was the pseudo-second order model (R^2^ ≥ 0.903) [85]. Studies on congo red and methylene blue removal using sludge and coconut shell char [63] and the removal of lead using COS with ammonium polyphosphate co-pyrolyzed char also support this trend by revealing that the kinetic adsorption data correlated more to the pseudo-second-order model [110]. The discussion above concludes that most reaction follows the pseudo-second order reaction proving the involvement of different adsorption sites on a solid substrate randomly colliding with each other during a rate-limiting mechanistic step [128]

Further information on kinetic models and their application will be detailed in Section 3.4.2. Table 6 shows the essential details regarding kinetic adsorption modelling for pollutant removal using co-pyrolyzed chars.

**Table 6:** Kinetic studies of water treatment using co-pyrolyzed chars

Feedstock	Pollutant	Experiment conditions	Best kinetic model	R^2^/ SSE	Parameter values	Reference
Inorganic pollutant removal studies						
SS and walnut shell	Ammonium Phosphate	Adsorbent amount 0.1 mg 50 mg/L- pollutant concentration Time interval: 1 to 50 h	Intraparticle diffusion Pseudo-second order	0.970 0.945	K_3_ = 0.299 C = 0.418 K_2_ = 0.022 q_e_ = 50.36	[113]
Nano-zero-valent iron and SS	Arsenic	Initial pollutant concentration: 20 mg/L Adsorbent amount: 4 g/L Contact time: 24 h Initial pH: 2	Liquid film diffusion	0.9491	q_e_ = 3.507 mg/g A_F_ = 0.7078, k_f_ = 0.2298 h^−1^	[89]
Molasses and dewatered alum sludge	Arsenic	Initial pollutant concentration: 300 mg/L, volume: 20 mL Adsorbent amount: 0.3 g Time: 8 h	Pseudo-second order	0.97	qe = 13.93 mg/g k_2_ = 0.4 × 10^–3^ g/mg min	[108]
Poplar bark (SB) and thiourea Poplar saw dust (MB) and thiourea	Cadmium	Initial pollutant concentrations:100 mg/L, volume: 20 mL Time interval: 5, 10, 30, 60, 120, 240, 360, 720, and 1440 min Adsorbent amount: 0.08 g (1), 0.10 g (2) pH: 7	Pseudo-second order	0.92 0.99	k_2_ = 0.0184 g/mg/min q_e=_ 12.19 mg/g k_2=_ 0.385 g/mg/min q_e_ = 0.652 mg/g	[91]
Cantaloupes straw and polypropylene	Cadmium	Adsorbent amount: 0.02 g Initial pollutant concentration: 150 mg/L Temperature- 25 ºC Time intervals: 0, 0.1, 0.25, 0.5, 1, 2, 4, 7, 12, 18, and 24 h	Pseudo-second order	0.984	q_e_ = 0.0268 mg/g K_2_ = 0.0303 g/mg min	[114]
SS and hazelnut shell	Copper	Initial concentrations: 55 mg/L Time interval: 0 min, 10 min, 30 min, 1 h, 2 h, 5 h, 10 h and 24 h Temperature- 25 °C	Pseudo-second order	0.9994	k_2=_ 43.75 mg/g h q_e_ = 0.652 mg/g	[116]
SS and hazelnut (magnetized with nanosized γ-Fe2O3)	Copper	Adsorbent amount: 1.25 g/L Initial pollutant concentration: 100 mg/L Contact time: 0, 30, 60, 120, 300, 600, 1440 min	Pseudo-second order	0.999	k_2=_ 0.04 mg/g h q_e_ = 71.43 mg/g	[115]
Nano-zero-valent iron and SS	Chromium	Initial pollutant concentration: 50 mg/L Adsorbent amount: 4 g/L Contact time: 24 h Initial pH: 2	Pseudo-second order	0.9994	q_e=_ 0.2383 mg/g k_2_ = 11.49 g/mg h	[88]
Sucrose with waste red mud	Chromium	Initial pollutant concentrations: 10 and 25 mg/L Adsorbent amount: 2 g/L, volume: 20 mL Temperature: 25 °C pH: 2.10 ± 0.05, ionic Shaking speed: 200 rpm Interval time: 5–180 min	Pseudo-second order	R^2^ = 0.990 R^2^ = 0.996	Initial: 10 mg/L qcal = 3.045 mg/g k_F_ = 0.007 mg/(g min1/2) Initial: 25 mg/L q_cal_ = 7.244 mg/g k_F_ = 0.004 mg/(g min1/2)	[59]
Sludge and corncobs (SCB)	Lead	Amount of adsorbent: 0.1 g Initial pollutant concentrations: 100 mg/L, volume: 25 mL Temperature: 25 ºC Time intervals: 0.5, 1, 2, 4, 8 h	Pseudo-second order SCB (300 °C) SCB (500 °C) SCB (700 °C)	R2 > 0.99	q_e_ = 24.8611 mg/g K_2_ = 0.0322 g/mg min q_e_ = 29.1886 mg/g K_2_ = 0.0322 g/mg min q_e_ = 27.9759 mg/g K_2_ = 0.0551 g/mg min	[98]
Halloysite and coconut shell	Lead	Adsorbent amount: 100 mg Initial pollutant concentration: 100 mL Temperature- 25 ºC Time intervals: 1 min, 3, 5, 10, 30, 60, 100, 200, 300, 400, 500, 600, 720, 1080, 1440 min	Pseudo-second order	0.999	q_e_ = 684.937 ± 2.633 mg/g K_2_ = 0.00011 ± 0.00002 g/gm min	[90]
Organic pollutant removal studies						
SS and bamboo waste	Ciprofloxacin	Adsorbent amount: 0.25 g Initial pollutant concentrations: 10 mg/L, volume: 100 mL Temperature: 30 ºC Interval times: 0 h, 1 h, 2 h, 5 h, 8 h, 12 h, 24 h	Pseudo-second order	0.99	Q_cal_ = 4.24 mg/g k_2_ = 0.63 g/mg h	[118]
Mixed date pits and olive stones	Dibenzothiophene	Adsorbent amount: 0.3 g Initial pollutant concentrations: 200 mg/L, volume: 25 mL Time intervals: 10–90 min Temperature: 50 °C	Pseudo-second order	0.9998	q_e_ = 16.10 mg/ g k_2_ = 0.035 mg/g min	[73]
Hematite-biochar composite (FOC) Pyrite-biochar composite (FSC)	Norfloxacin	Adsorbent amount:0.1 g Initial pollutant concentrations: 2 mg/L to 30 mg/L Time interval: 0 to 250 min pH: 7.0 ± 0.05	Pseudo-second order	0.9990 0.9995	Q_cal_ = 1.661 mg/g k_2_ = 0.1099 g/mg min Q_cal_ = 1.973 mg/g k_2_ = 0.2251 g/mg min	[93]
Coal tar pitch and vinasse	Phenol	Adsorbent amount: 0.025 g Initial pollutant concentrations: 50 mg/L Temperatures: 25 ºC	Pseudo-second order	0.9996	q_e=_ 39.4 mg/g k_2 =_ 0.0009 g/mg min	[59]
Corn straw and sawdust Two co-pyrolyzed chars (1:1) at 300 ºC (BC_300A_) and 800 ºC (BC_800A)_	Atrazine	Adsorbent amount: 20 mg Initial pollutant concentration: 25 mg/L Temperature: 25 ºC	Pseudo-first order	0.987 0.961	q_e,cal_ = 6.76 ± 0.0763 mg/g k_1_ = 1.07 ± 0.0628 h^−1^ q_e,cal_ = 5.23 ± 0.0855 mg/g k_1_ = 2.43 ± 0.2407 h^−1^	[119]

#### Temperature and thermodynamic analysis

As previously mentioned, temperature plays a key role in the adsorption process. In order to conduct the temperature and thermodynamic analysis, the isotherm parameters found at different temperatures after modelling are used to calculate the Gibbs free energy after calculating the thermodynamic equilibrium constant. Finally, the Van’t Hoff plot is constructed to understand and evaluate the changes in entropy and enthalpy [100]. Table 7 shows nine studies, which focused on the thermodynamic analysis for the adsorption of pollutants from water using co-pyrolyzed chars (some activated, some non-activated). The table contains ΔGº, ΔH º, and ΔSº values all calculated from the above-mentioned method.

**Table 7:** Thermodynamic studies of water treatment using co-pyrolyzed chars

Feedstock char	Pollutant	Temperature (K)	ΔGº (kJ/mol)	ΔH º (kJ/mol)	ΔSº kJ/ (mol.K)	Remarks	Reference
Sucrose and red mud	Chromium	298 308 318	-3.258 -6.545 -9.779	89.02	0.297	Spontaneous, chemical sorption, increase in system irregularity	[59]
SS and tea waste	Cadmium	298 308 318	-0.9479 -1.2971 -1.8200	32.3036	122.6315	Spontaneous, physical sorption, increase in system irregularity	[93]
SS and rice straw	Chromium	283 298 313	-0.9 -1.6 -2.7	15.8	58.9	Spontaneous, physical sorption, increase in system irregularity	[97]
SS and walnut shell	Ammonium Phosphate	298 303 308 313 318 298 303 308 313 318	6.35 6.89 7.35 8.11, 8.62 − 1.47 − 2.35 − 3.15 − 4.11 − 3.43	− 27.93 36.39	-0.115 0.128	Non-spontaneous, physical sorption, decrease in system regularity Spontaneous, physical sorption, increase in system irregularity	[113]
Hematite-biochar composite Pyrite-biochar composite	Norfloxacin	288.15 298.15 308.15 288.15 298.15 308.15	− 12.9345 − 15.7507 − 17.2404 − 20.5437 − 20.3434 − 19.7422	49.2746 − 31.9914	0.2168 − 0.0396	Spontaneous, physical sorption, increase in system irregularity spontaneous, physical sorption, decrease in system regularity	[111]
Nano-zero-valent iron and SS	Chromium	288 298 303 313 318	-5.947 -6.529 -7.156 -8.308 -11.43	41.93	163.8	Spontaneous, chemical sorption, increase in system irregularity	[88]
Coal tar pitch and vinasse	Phenol	288.15 (10 ppm) 298.15 308.15 288.15 (20 ppm) 298.15 308.15 288.15 (30 ppm) 298.15 308.15 288.15 (50 ppm) 298.15 308.15 288.15 (70 ppm) 298.15 308.15	-9.2 -9.5 -9.8 -11.3 -11.7 -12.1 -8.9 -9.2 -9.6 -8.3 -8.6 -8.9 -8.0 -8.3 -8.6	2.6 6.7 2.8 3.0 3.2	31.9 39.3 31.0 29.0 27.9	Spontaneous, physical sorption, increase in system irregularity	[111]
Nano-zero-valent iron and SS	Arsenic	298 303 308 313 318	-5047.4 -6304.4 -6922.0 -8302.6 -9754.6	63.03	228.2	Spontaneous, chemical sorption, increase in system irregularity	[89]
SS and coconut shell	Methylene blue Congo red	**One-stage** 283.15 298.15 313.15 **Two-stage** 283.15 298.15 313.15	-16.95 ± 0.15 -20.39 ± 0.22 -23.12 ± 0.14 -19.77 ± 0.15 -23.65 ± 0.15 -27.31 ± 0.13	40.02 ± 2.39	201.10 ± 8.02	Spontaneous, chemical sorption, increase in system irregularity	[63]

The values give information regarding the adsorption system. Negative ∆G values indicate that the adsorption process is spontaneous; furthermore, it is also proposed that a ∆H value less than 80 kJ/mol represents a physical adsorption system [59]. Alternatively, a higher ∆H value generally between 80 and 400 kJ/mol implies a chemical adsorption system [59]. The results of this study can be seen in Table 7, using red mud and sucrose for chromium removal. The higher the negative value, the more spontaneous is the reaction (observed with an increase in temperature). A similar trend was also observed in a study by Fan et al. (2018) utilizing SS and tea leaf chars for the removal of cadmium from water as shown in Table 7. The ∆H values between 80 and 400 kJ/mol suggest an endothermic process with chemical adsorption taking place [59]. Finally, a positive ∆S value indicates increased randomness and disorder at the adsorbate-adsorbent interface. The results of the study revealed a ∆H value lesser than 80 kJ/mol and a positive ∆S value indicating physical adsorption [59]. The remarks of the other studies in Table 7 are based on this study.

### Adsorption mechanisms

Some studies regarding pollutant adsorption mechanisms of co-pyrolyzed chars will be discussed in this section. A study used Fourier-transform infrared spectroscopy (FTIR), X-ray powder diffraction (XRD), scanning electron microscopy with energy-dispersive X-ray analysis (SEM–EDX), and zeta potential results analyses to confirm adsorption mechanisms, including electrostatic attraction, complexation, and precipitation involved in the removal of chromium by co-pyrolyzed sucrose and waste red mud. FTIR results revealed that -OH, C = O, and C-O bands had completely disappeared potentially due to the surface complexation with the chromium ions [59]. Additionally, XRD revealed that there was a presence of Cr_2_FeO_4_ and Cr_2_O_3_ peaks displaying the redox reaction between iron in the adsorbent and chromium ions (which then precipitated to the adsorbent surface). Furthermore, SEM–EDX analysis also supported the potential mechanism that drove the adsorption process.

The next study concluded ion exchange to be the removal mechanism for chromium by co-pyrolyzed rice waste and polyethylene [59]. Upon ion analysis of the wastewater after adsorption, an increase in the cation concentration (K = 24.7 ppm) and a decrease in the chromium concentration (72.7 ppm) from wastewater was observed; this study proved that most of the chromium is removed by ion exchange with potassium. Therefore, the pore characteristics also must have played a key role in pollutant removal.

Furthermore, another study focused on understanding the mechanism of removal of lead using chars produced from co-pyrolyzing skins, pith, and leaves with polyethylene. The characterization studies, applying FTIR, XRD, SEM, zeta potential analysis, concluded that the adsorption mechanisms were taking place in the form of Pb–O or hydroxyl binding in addition to ion exchange. Since the biochar was activated by KOH, the adsorption resulted in the precipitation of K_2_Pb_2_O_3_ via complexation. The activation also brought some hydroxyl groups leading to the formation of Pb_3_(CO3)_2_(OH)_2_ and additionally, the carbonate from the char bonded with lead to form PbCO_3_. These results were in accordance with the experiments results obtained at the end of the study as well [67].

A similar study mainly focused on FTIR, XRD, and SEM–EDX to understand the mechanism for the removal of chromium by nZVI and SS co-pyrolyzed char [88]. The mechanism mainly involved the majority of the Cr (VI) being directly removed by the magnetic biochar, some getting reduced to Cr (III) and then removed by adsorption. The electrons were supplied by Fe^2+^, Fe^0^ and the organics in the produced char. Further modelling also revealed that the rate-controlling steps for chromium removal were liquid-film/intra-particle diffusions and liquid-film diffusion/chemical reaction processes. A follow-up study was conducted using the same co-pyrolyzed char to remove arsenic. Similar to the previous research, the potential mechanism was aligned to a liquid film diffusion model based on chemical reaction as arsenic was immobilized on the co-pyrolyzed char by speciation of As-O-Fe. The removal rate of arsenic based on all formerly mentioned analytical tools proved that the mechanism involved active participation of iron-containing substances and organics in the char in the removal process [89].

A further study [93] analyzed the removal of cadmium using SS and tea waste co-pyrolyzed char by FTIR analysis. The peaks that reduced or disappeared after adsorption concluded that the following functional groups: –OH, –C = C or C = O, –CO, –CH aided in the binding of cadmium to the co-pyrolyzed char through ion exchange, surface complexation, and cation-p interaction.

**Fig. 3:**
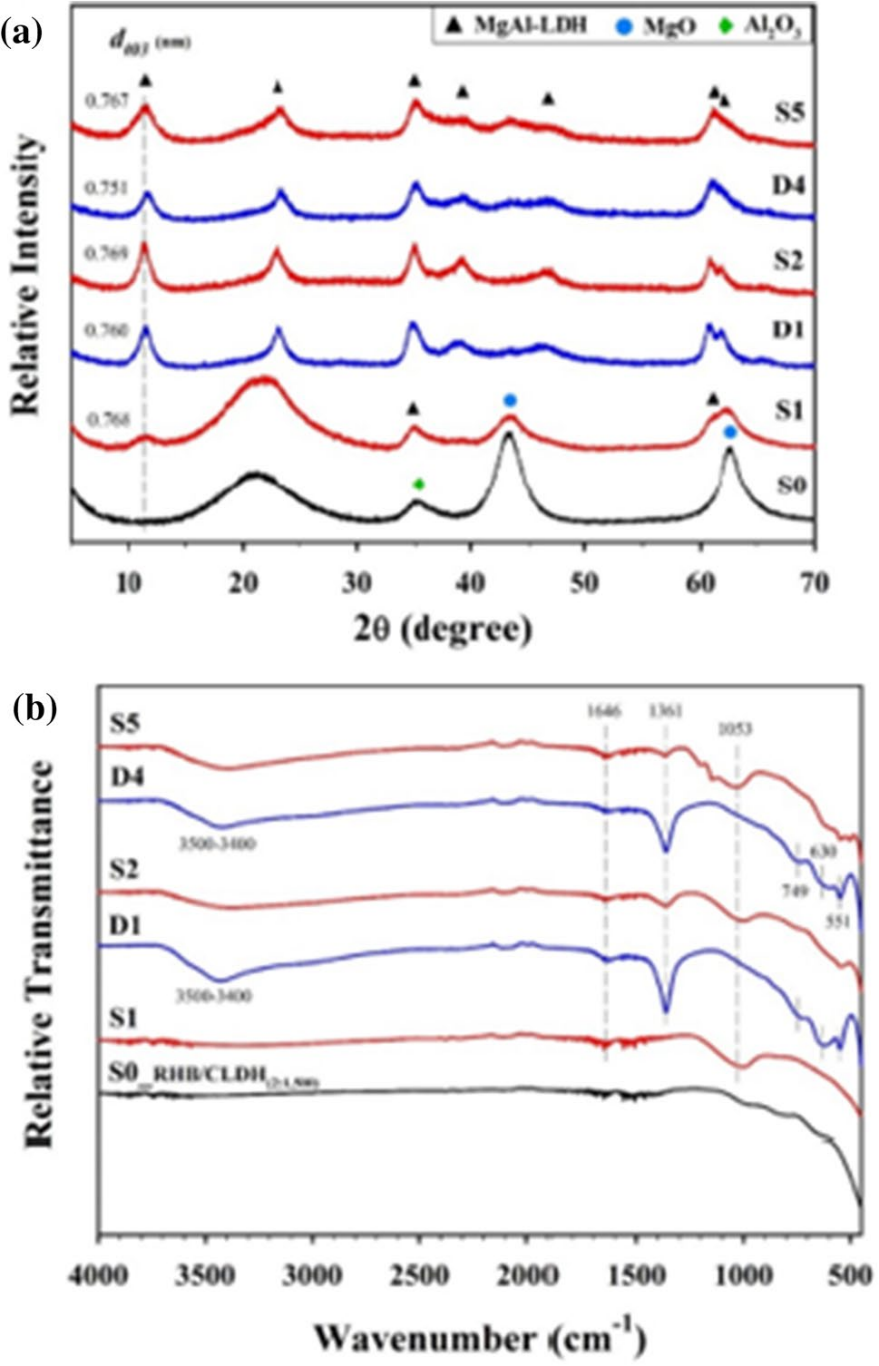
Before and after adsorption (**A**) XRD spectra. (**B**) FTIR spectra

**Fig. 4:**
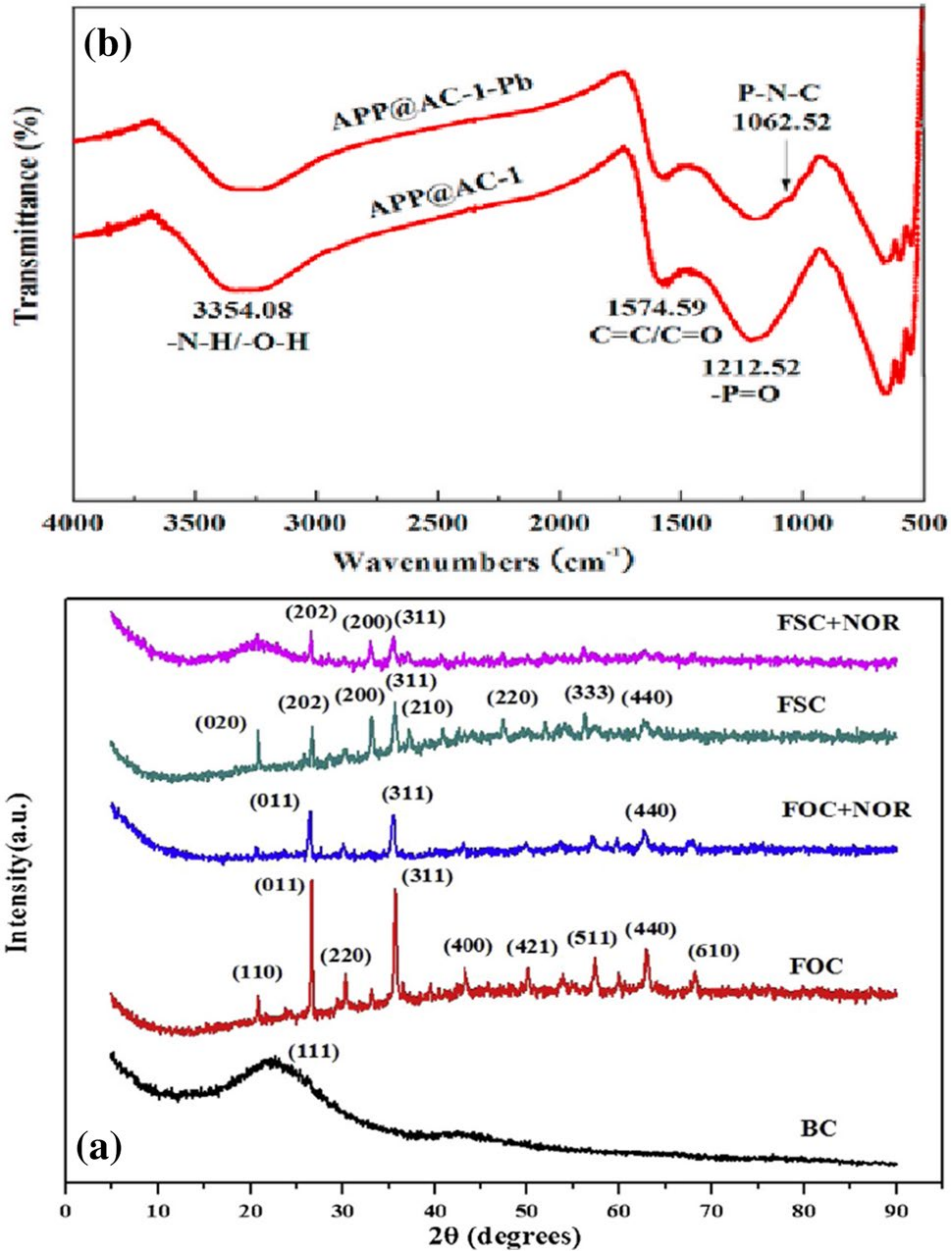
FTIR spectra of fresh and used co-pyrolyzed char

**Fig. 5:**
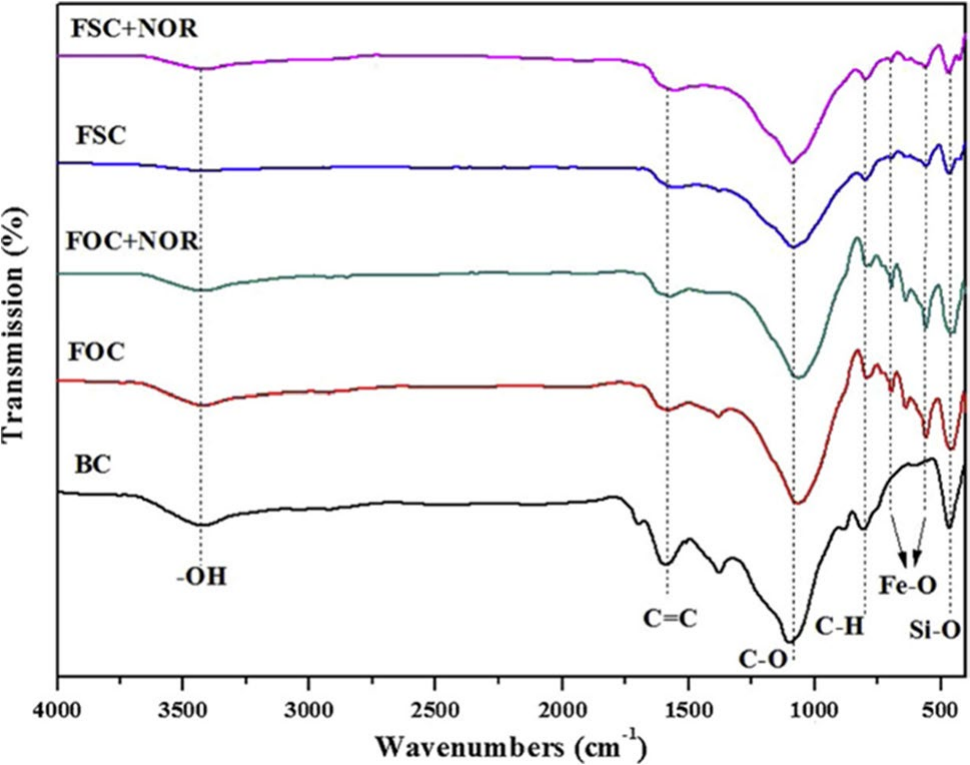
Before and after adsorption (**A**) XRD spectra (**B**) FTIR spectra

**Fig. 6:**
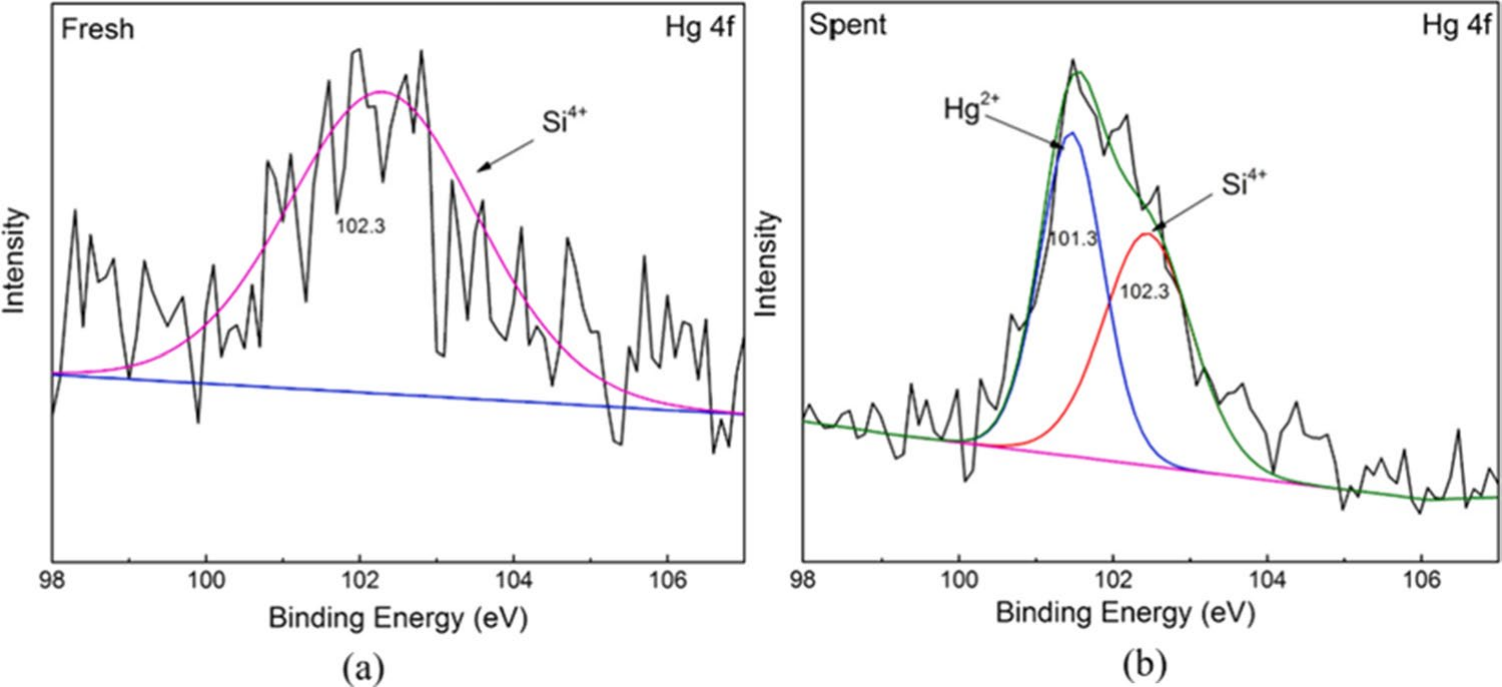
XPS spectra of fresh and used co-pyrolyzed char

Table 8 presents and reviews the key features of four other co-pyrolysis adsorption mechanism studies using molecular analytical tools (Figs. 3, 4, 5, and 6). As seen in the previous studies, analytical tools such as FTIR, XRD, SEM, in addition to XPS (X-ray photoelectron spectroscopy) usually represent what is taking place during adsorption. Understanding the mechanism will help design further experiments and application of these adsorbents.

**Table 8:** Proposed adsorption mechanisms of water treatment using co-pyrolyzed chars

Feedstock	Suggested mechanism	Analytical tool	Analysis result	Reference
Rice husk with Mg/Al-calcined layered double hydroxides	The FTIR and XRD results showed a strong P-O bond which reveals a strong indication between the char and phosphate by ligand exchange in the form of monodentate and bidentate inner-sphere surface complexes	XRD FTIR	See Fig. 3	[87] Copyright, 2019, Elsevier
COS and polyphosphate	The modified char showed multiple functional groups in the co-pyrolyzed char including –NH/-OH, C + C/C = O, P = O, P–N–C, and P–O–C, and these spectra also reveal that the bands associated with -N–H/-O–H, –C = O, -O-C = O, and -O–P–O groups of the char shifted and the areas below it decreased The increased functional groups sped up the adsorption process and high adsorption capacities	FTIR	See Fig. 4	[110] Copyright, 2020, Elsevier
Pyrite-biochar composites (FSC) than hematite-biochar composites (FOC)	The FTIR spectrum analysis showed that both the chars had a slight shift after adsorption of norfloxacin specifically at 3426, 1588, 1099, 807, and 465 cm^−1^, corresponding to -OH, C = C, C-O, C-H, and Si–O) — the reduction in C = C, C-O, C–C peaks indicates the π–π connections between norfloxacin and the chars The XRD analysis after adsorption revealed that the difference in peaks was only small, concluding good stability	FTIR XRD	See Fig. 5	[111] Copyright, 2019, Elsevier
Bamboo and bromine flame retardant	The XPS spectra of the co-pyrolyzed char before and after the removal of mercury by the co-pyrolyzed char — results revealed a strong peak at 102.3 ± 0.2 eV, signifying Hgº for the fresh char. However, a new peak representing Hg^2+^ was found instead for the used char Additionally, another peak representing C–Br decreased after adsorption, signifying that Br- converted to Hg0 after adsorption	XPS	See Fig. 6	[96] Copyright, 2021, Elsevier

### Regeneration studies

Concerning the study of reusability of the chars, regeneration experiments focus on repeated cycles of adsorption and desorption to achieve greater economic use from the char and environmental sustainability. Several chemicals including nitric acid, sulfuric acid, hydrochloric acid, chelating agents like EDTA, and distilled water are usually considered [129]. Literature studies using some of these eluents and a few others are discussed.

A study on chromium adsorption by red mud and sucrose co-pyrolyzed chars assessed the use reusability of the magnetic biochar [59]. During desorption, a range of reagents was used for elution experiments using NaOH, Na_2_CO_3,_ HNO_3,_ demonstrating desorption performances of 44.6%, 20.0%, and 3.9%, respectively. It was also found that the adsorbed chromium was stable on the char in both acidic and neutral conditions. All three adsorption and desorption rounds showed no change in the adsorption capacity, although, at the end of the fourth cycle, a 9% reduction in removal efficiency was detected [59].

Another study conducted five cycles of adsorption and desorption using co-pyrolyzed rice straw and SS char using NaOH elution [97]; unlike the previous study, there was a reduction in the removal efficiencies at each cycle. The first cycle removed 90.8% of chromium. The subsequent four cycles removed 79.1%, 70.8%, 66.2%, and 61.3%, individually. The steady decrease in the removal percentages was attributed to a reduction in the adsorbent and pore structures in addition to irreversible saturation of adsorption sites. However, the removal rate, 61.3%, remained a fixed constant in the following cycles, proving a good reusability feature of the char [97]. Another study using rice husk (RH)-derived co-pyrolyzed biochar with Mg/Al-calcined layered double hydroxide chars for the removal of phosphate conducted 5 cycles of adsorption and desorption using NaOH as an eluent. They demonstrated similar results to the previous study [85]. There was a steady decrease in the removal percentages remaining just above 80% even after 5 cycles, speculated to be due to the replacement of small anions like OH or CO_3_^2−^ for phosphate ions. Similar promising results were shown by halloysite and coconut shell co-pyrolyzed char even after four cycles of lead adsorption–desorption with removal rates greater than 95% [90]

One study considered the removal of norfloxacin using hematite-biochar composites (FOC) and pyrite-biochar composites (FSC) by conducting five cycles of adsorption and desorption using methanol as the eluent [72]. The results demonstrated a constant decrease in the pollutant removal plateauing at 75% for FOC and 83% for FSC after several cycles. The recovery of adsorbents was found to be 90% after each of the cycles. This work concluded the good regeneration and recycling capabilities of the adsorbents [111]. Furthermore, a study on the regeneration of co-pyrolyzed COS and ammonium polyphosphate showed reduced removal rates with an increasing number of cycles with sodium acetate elution and a removal capacity of 74.2 mg/g stabilized after four cycles [110].

The activated char produced from olive stones and mixed date pits was used to remove dibenzothiophene by five consecutive cycles of adsorption and desorption using n-hexane as the eluent. The first cycle removed 90.01%, which reduced to 85.02% during the final cycle, therefore showing good elimination of DBT due to the presence of active sites even at the end of the fifth cycle [73]. Some other studies show relatively lower removal efficiencies (between 40 and 60%) after 4 cycles of copper adsorption–desorption by co-pyrolyzed chars [89, 118].

**Fig. 7:**
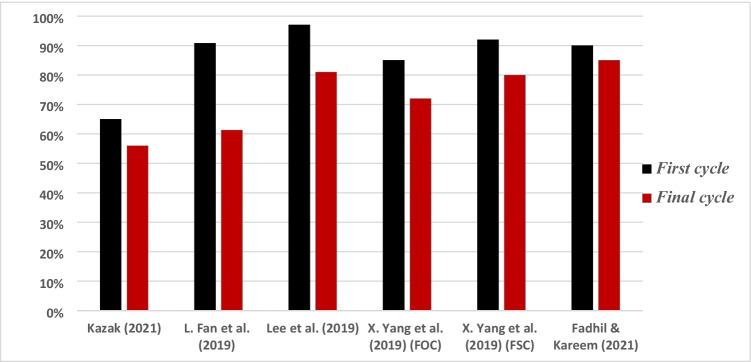
Regeneration studies of water treatment using co-pyrolyzed chars

Figure 7 shows the approximate removal efficiencies (first and final cycles) of some of the studies discussed above. Regeneration studies are essential to know the reusability of the adsorbent, making it more sustainable.

### Real water studies

It is imperative to conduct detailed studies on how co-pyrolyzed chars perform on real water samples and not just synthetic waters. To date, only two studies focused on such work while utilizing co-pyrolyzed chars for water treatment. The first study focused on the removal of chromium using co-pyrolyzed red mud and sucrose considering the same initial concentration as the isotherm analysis providing the best conditions, which were 2 g/L of adsorbent, 2.10 ± 0.05 of initial pH, contact time of 120 min, and a shaking speed of 200 rpm at 25 °C. The results aligned with the synthetic chromium solution demonstrating 20.41 mg/g adsorption capacity. This proved that the char applies to real water samples without any adverse effect [59]. On the other hand, a study using pyrolyzed chars of wood and plastic wastes for aquaculture wastewater treatment found the phosphate and turbidity removal were highest with removal percentages of 86.36% and 78.25% respectively (for the non-activated biochar) [130]. In general, this study also concluded no significant difference in removal rates of pollutants between activated and non-activated char samples.

## Challenges and future prospects

Research activities related to water treatment using co-pyrolyzed chars have some challenges. As a whole, research conducted on co-pyrolysis focuses more on fuel production rather than chars; therefore, more studies are needed to focus on the production of adsorbents that can be used for water treatment. Furthermore, more research needs to be focused on the co-pyrolysis of biomass with other wastes like plastics as a representation of municipal wastes together. Optimizing the ratios and understanding the effect of mixing on pyrolysis and its product distributions is essential for successful application. This sustainable approach will reduce the time and energy consumed compared to the individual pyrolysis [22]. It would also be interesting to compare the quality of chars and adsorption capacities of co-pyrolyzed waste and mixed single biochars produced under the same conditions [22]. There is scope for testing different variables when it comes to co-pyrolysis, including the particle size, heating rate, and nitrogen gas flow rate.

Furthermore, adsorption capacities of the adsorbents described in Section 2.1 can be significantly improved by activation to be more applicable for water treatment [12]. There is a need to explore and compare more activation techniques to find the most suitable method. This will also vary depending on the biochar feedstock type and the water pollutant removal application.

Regarding water treatment, understanding the bonding mechanisms of the pollutants in addition to understanding the condition of the adsorbents before and after treatment is crucial for further application — very few current studies focus on this. Similarly, recent review papers on utilizing treated wastes for water treatment suggest more research on the desorption and regeneration of the used adsorbents [22, 131, 132]. The recovery of precious pollutants like heavy metals is also recommended following a sustainable and cyclic economic pathway [88]. Most importantly, for effective adsorbent testing, wastewater effluents [3] comprising the more typical multicomponent mixture of pollutants need to be tested [88] rather than simulated water containing a single pollutant. Furthermore, emphasis should be given to solving the problems on removing emergent contaminants that are harmful even at low concentrations [131].

In recent times, especially in the era of COVID-19, plastics and microplastics are receiving a lot of attention as pollutants. There are several studies discussing the use of biochar for the removal of microplastics. One such study focused on pyrolyzed and steam activated pine bark and spruce bark samples showing good removal of large microplastic particles [133]. Another study showed excellent polystyrene microplastic removal capabilities when using magnetic sawdust biochars facilitated by electrostatic and chemical bonding interactions [134]. However, more research on using co-pyrolyzed char for the same application needs to be conducted.

An adsorbent that is versatile and can remove both organic and inorganic pollutants will also be advantageous [132]. This is potentially achievable since certain co-pyrolyzed biomass adsorbents are known to adsorb both classes of compounds (Section 2.2). Designing a reactor for these modern adsorption applications [132] for large-scale/pilot scale studies needs to be performed using the actual industrial conditions [131], for example, contaminant mix and the actual pollutant concentrations, which are mostly not the same as the individual isotherm q_max_ due to competition from contaminants. Finally, there is a need to address the lack of techno-economic analysis data required for employing and implementing the use of these adsorbents in water treatment. Hence, there is an urgent need to address this in research, using application of process simulation studies to investigate the effect of varying parameters.

## Conclusion

The review summarizes recent co-pyrolysis studies of biomass with other feedstock such as lignocellulosic wastes, plastics, and some other biomass. It is a comprehensive review of experimental studies focusing a whole section on the activation of co-pyrolyzed chars for producing better quality activated chars/activated carbons for water treatment (reporting the surface area and pore volume when provided and the adsorption capacities). Very attractive studies have been reported — one study on co-pyrolyzed cyanobacteria and plastics demonstrated a surface area of 2135 m^2^/g upon alkali activation with K_2_CO_3._ Furthermore, the effects of various parameters including pH, temperature, contact time, and char dosage have been analyzed. Section 2.2.3 discussed research conducted using isotherm models, kinetic models, and thermodynamic analysis. These studies provide significant, although sometimes limited, insights on mechanisms and rates regarding how co-pyrolyzed chars can be used as adsorbents for water treatment purification. Furthermore, the need for further research into adsorption mechanisms, regeneration or reusability, and real water studies to understand the actual applicability of co-pyrolyzed chars has been described. Most studies discussed in this paper favored the Freundlich model for isotherm equilibrium modelling and the pseudo second-order model for the kinetic modelling; a wider range of models are required to enhance the accuracy of the design parameters used for the treatment system design. Additionally, the thermodynamic analysis showed that most adsorption reactions are spontaneous, and the analytical analysis using FTIR, XPS, SEM, and XRD provides good evidence for the adsorption mechanisms. The few studies on reusability studies show that the chars can be used for at least 4 to 5 regeneration cycles and that regeneration typically plateaus.
